# Durable Disease Control With Atezolizumab Plus Bevacizumab but Limited Post‐Progression Outcomes: A Multicenter Real‐World Study in Advanced Hepatocellular Carcinoma

**DOI:** 10.1002/cam4.71655

**Published:** 2026-02-18

**Authors:** Masanori Inoue, Sadahisa Ogasawara, Kazuhisa Asahara, Rui Sato, Shunji Watanabe, Haruka Anzai, Kazuo Tsubura, Taro Watabe, Makoto Fujiya, Teppei Akatsuka, Ryo Izai, Keiichi Katayama, Midori Sawada, Takahiro Tsuchiya, Chihiro Miwa, Ryohei Yoshino, Takuya Yonemoto, Kentaro Fujimoto, Hidemi Unozawa, Sae Yumita, Hiroaki Kanzaki, Keisuke Koroki, Kazufumi Kobayashi, Masato Nakamura, Naoya Kanogawa, Shingo Nakamoto, Masayuki Yokoyama, Jun Kato, Michihisa Moriguchi, Takeshi Aramaki, Naoki Morimoto

**Affiliations:** ^1^ Department of Gastroenterology, Graduate School of Medicine Chiba University Chiba Japan; ^2^ Division of Interventional Radiology Shizuoka Cancer Center Shizuoka Japan; ^3^ Division of Gastroenterology, Department of Medicine Jichi Medical University Tochigi Japan; ^4^ Biostatistics Division, Center for Research Administration and Support National Cancer Center Tokyo Japan; ^5^ Department of Gastroenterology and Hepatology Kyoto Prefectural University of Medicine Kyoto Japan

**Keywords:** atezolizumab, bevacizumab, duration of disease control, duration of response, hepatocellular carcinoma, second line

## Abstract

**Introduction:**

Atezolizumab plus bevacizumab (Atez/Bev) has become the standard first‐line therapy for advanced hepatocellular carcinoma (HCC), but optimal second‐line strategies remain unclear. This multicenter retrospective study compared survival outcomes and tumor control among first‐line regimens and evaluated post‐progression efficacy.

**Methods:**

We retrospectively analyzed 1542 patients with advanced HCC who received sorafenib (SOR), lenvatinib (LEN), or Atez/Bev between 2009 and 2022. The main cohort (*n* = 612) excluded patients who underwent concomitant or inter‐line locoregional therapy or entered clinical trials after first‐line treatment. Overall survival (OS), progression‐free survival (PFS), post‐progression survival (PPS), duration of disease control (DDC), and duration of response (DOR) were estimated by Kaplan–Meier analysis and compared by the log‐rank test. The impact of disease control and objective response on OS was assessed using time‐dependent Cox models.

**Results:**

Median OS was 15.4 months for SOR, 13.1 for LEN, and 20.6 for Atez/Bev (*p* = 0.002); Median PFS was 3.5, 5.7, and 7.5 months, respectively (*p* < 0.001); and median PPS was 10.8, 6.4, and 9.0 months, respectively (*p* = 0.049). Atez/Bev achieved the longest DDC (8.5 months) and DOR (22.1 months). Disease control and objective response were associated with improved OS (HR: 0.51/0.42 for SOR, 0.54/0.62 for LEN, 0.34/0.35 for Atez/Bev). After progression, median second‐line PFS was 5.5 months for SOR, 2.5 for LEN, and 3.0 for Atez/Bev (*p* = 0.001).

**Conclusion:**

Atez/Bev provided superior survival and durable tumor control; however, post‐progression outcomes remained unsatisfactory, underscoring the need for improved second‐line approaches after Atez/Bev failure.

**Abbreviations**
AE: adverse event
AFP: alpha‐fetoprotein
ALBI: albumin–bilirubin
Atez/Bev: atezolizumab plus bevacizumab
BSC: best supportive care
CAB: cabozantinib
CI: confidence interval
CR: complete response
DCR: disease control rate
DDC: duration of disease control
DOR: duration of response
ECOG PS: Eastern Cooperative Oncology Group performance status
EHS: extrahepatic spread
HAIC: hepatic arterial infusion chemotherapy
HBV: hepatitis B virus
HCC: hepatocellular carcinoma
HR: hazard ratio
ICI: immune checkpoint inhibitor
LEN: lenvatinib
MVI: macrovascular invasion
NA: not available
ORR: objective response rate
OS: overall survival
PD: progressive disease
PFS: progression‐free survival
PPS: post‐progression survival
PR: partial response
RAM: ramucirumab
RECIST: Response Evaluation Criteria in Solid Tumors
REG: regorafenib
SOR: sorafenib
STRIDE: single tremelimumab regular interval durvalumab
TACE: transarterial chemoembolization
TKI: tyrosine kinase inhibitor
VEGF: vascular endothelial growth factor
VEGF‐TKI: vascular endothelial growth factor–tyrosine kinase inhibitor

## 1 Introduction

Systemic therapy for advanced hepatocellular carcinoma (HCC) has profoundly transformed with the advent of combination immunotherapies. The first to establish this paradigm was atezolizumab plus bevacizumab (Atez/Bev), which demonstrated a clear overall survival (OS) advantage over sorafenib (SOR) in the phase III IMbrave150 trial (median OS: 19.2 vs. 13.4 months), marking the beginning of combination immunotherapy in advanced HCC [1]. Since then, other regimens such as durvalumab plus tremelimumab (STRIDE) and nivolumab plus ipilimumab have also shown clinical benefits, demonstrating superiority over existing vascular endothelial growth factor–tyrosine kinase inhibitors (VEGF‐TKIs) in randomized phase III trials [2, 3]. Nevertheless, Atez/Bev remains the cornerstone of first‐line therapy, having been rapidly approved across the globe and widely adopted in routine practice, with real‐world studies consistently reproducing the trial findings and confirming durable benefits in OS and progression‐free survival (PFS) [4, 5, 6].

A distinguishing feature of immune checkpoint inhibitor (ICI)‐based combinations is their ability to induce deep and durable responses in a subset of patients. This is in contrast to the transient tumor control typically achieved with molecular targeted agents. This phenomenon is already well recognized in other tumor types treated with ICIs [7, 8]. This property has drawn attention to surrogate endpoints that capture the quality and persistence of tumor control [9, 10]. The duration of response (DOR) has emerged as a candidate predictor of long‐term benefit and has shown strong associations with OS in post hoc analyses of the IMbrave150 trial [11]. However, immunotherapy does not benefit all patients; a substantial proportion either fail to respond or experience early progression. Outcomes for these non‐responders remain poorly described [1, 2, 3, 7, 8]. For such patients, endpoints beyond first‐line therapy, including post‐progression survival (PPS) and the efficacy of subsequent systemic therapies [12, 13], are clinically critical but remain underexplored in HCC. To date, most studies have focused on the OS and PFS of front‐line combination immunotherapies, with limited data on outcomes beyond progression, particularly PPS, and second‐line efficacy.

These knowledge gaps are particularly important in the post‐Atez/Bev setting, where no standard second‐line strategy has been established. In routine practice, VEGF‐TKIs such as sorafenib (SOR), lenvatinib (LEN), regorafenib (REG), ramucirumab (RAM), and cabozantinib (CAB) are commonly used after Atez/Bev progression [14, 15, 16, 17]. However, none of these regimens have been validated in large prospective randomized trials, specifically in this setting, and most available evidence relies on retrospective series, small‐sample prospective studies, or narrative reviews [18, 19, 20, 21, 22, 23, 24]. Even comprehensive network meta‐analyses have not directly evaluated second‐line treatments after Atez/Bev [25]. Consequently, the optimal sequencing of systemic therapy and the extent to which post‐progression outcomes can be improved compared to earlier treatment eras remain uncertain. To date, there have been very few reports that comprehensively address treatment strategies after Atez/Bev, making it difficult to gain a true understanding of this increasingly common clinical scenario.

To address these challenges, we performed a multicenter retrospective cohort study of 1542 patients with advanced HCC treated with first‐line SOR, LEN, or Atez/Bev. The primary aim of this study was to clarify how Atez/Bev transformed treatment dynamics compared to prior VEGF‐TKI‐based standards. Whereas the TKI era was defined by only modest gains in OS and PFS without durable responses, Atez/Bev has introduced the prospect of deep, sustained tumor control and even treatment conversion. At the same time, its use has created new uncertainties regarding optimal post‐Atez/Bev strategies, highlighting the need for a comprehensive understanding of therapy sequencing in the immunotherapy era, based on real‐world data from a large multicenter cohort.

## 2 Methods

### 2.1 Patients and Study Design

This multicenter retrospective study included 1542 patients with advanced HCC who received first‐line systemic therapy with SOR, LEN, or Atez/Bev between June 2009 and March 2022. Data were collected from Chiba University Hospital, Kyoto Prefectural University of Medicine, Shizuoka Cancer Center, and Jichi Medical University Hospital, and integrated into a unified analysis dataset. The study protocol was approved by the ethics committees of all the participating institutions (Chiba University approval number: M10292). In accordance with the Japanese Ethical Guidelines for Medical and Health Research Involving Human Subjects, the requirement for written informed consent was waived, and an opt‐out approach was adopted. This manuscript was prepared with the assistance of ChatGPT, a language model developed by OpenAI that was used to refine the language. The authors have reviewed, revised and approved the final content.

All patients were categorized into four treatment eras based on drug approval timelines: Era 1 (June 2009–June 2017), predominantly SOR monotherapy prior to approval of second‐line agents; Era 2 (July 2017–March 2018), after approval of regorafenib (REG) enabling sequential therapy; Era 3 (April 2018–September 2020), after LEN approval and widespread use; and Era 4 (October 2020–March 2022), after Atez/Bev approval. The follow‐up data cut‐off was December 31, 2023. For the main analysis cohort (*n* = 612), patients from Era 1, those receiving concomitant or inter‐line locoregional therapy (e.g., transarterial chemoembolization [TACE], hepatic arterial infusion chemotherapy [HAIC]), and those enrolled in clinical trials after first‐line therapy were excluded (Figure S1).

### 2.2 Treatments

SOR was administered orally at 400 mg twice daily, and LEN at 12 mg once daily for patients weighing ≥ 60 kg, or 8 mg once daily for those weighing < 60 kg. Initial dosing generally followed the package insert, but in clinical practice, both agents were frequently subject to dose reduction or temporary interruption in response to adverse events (AEs). In some cases, treatment was initiated with a reduced dose at the physician's discretion.

Atez/Bev was administered intravenously every 3 weeks at 1200 mg of atezolizumab plus 15 mg/kg of bevacizumab. In cases of bevacizumab‐related AEs, bevacizumab was discontinued, while atezolizumab monotherapy was continued. If AEs were attributed to atezolizumab or both agents were deemed intolerable, the entire regimen was discontinued.

Treatment discontinuation occurred for several reasons: (1) radiological or clinical disease progression, (2) intolerable AEs, (3) deterioration of the general condition, or (4) patient request. In addition, treatment could be intentionally discontinued in favorable scenarios, such as achievement of confirmed complete response (CR) or transition to potentially curative therapies (e.g., surgical resection or ablation), representing proactive treatment‐off decisions.

### 2.3 Clinical Parameters and Radiological Assessment

Baseline variables included age, sex, etiology of liver disease, Child–Pugh class, albumin–bilirubin (ALBI) grade, Eastern Cooperative Oncology Group performance status (ECOG PS), tumor number, presence of macrovascular invasion (MVI), presence of extrahepatic spread (EHS), alpha‐fetoprotein (AFP) levels, and first‐line regimen. Prognostic data included the best overall response, date of progression, date of death, and last follow‐up. Data on post‐first‐line treatment regimens and progression dates were also collected.

Radiological assessment was conducted using dynamic contrast‐enhanced computed tomography or magnetic resonance imaging performed within 4 weeks prior to treatment initiation as baseline imaging. Tumor response was evaluated according to the Response Evaluation Criteria in Solid Tumors (RECIST) version 1.1. Imaging assessments were typically repeated every 4–8 weeks, reflecting the standard practice intervals in real‐world clinical settings, following each institution's protocol. When disease control was maintained and treatment was stable, extension of the imaging interval up to 12 weeks was permitted.

### 2.4 Statistical Analysis

The primary endpoint was overall survival (OS), defined as the time from initiation of first‐line therapy to death from any cause; patients alive at the data cut‐off were censored at the date of the last follow‐up. The secondary endpoints included objective response rate (ORR), progression‐free survival (PFS), post‐progression survival (PPS), duration of disease control (DDC), duration of response (DOR), and second‐line PFS (2nd PFS). PFS was defined as the time from first‐line treatment initiation to radiological progression or death, whichever occurred first. PPS was defined as the time from radiological progression during first‐line therapy to death. DDC was defined as the time from the first documentation of disease control (complete response [CR], partial response [PR], or stable disease [SD]) to progression or death, and DOR was defined as the time from the first documentation of CR or PR to progression or death.

Survival curves for OS, PFS, PPS, DDC, and DOR were estimated using the Kaplan–Meier method and compared using the log‐rank test. The association between disease control and objective response on OS was evaluated using time‐dependent Cox proportional hazards models, in which disease control or objective response was incorporated as a time‐dependent covariate (0 = not achieved; 1 = achieved). Analyses were conducted separately for the SOR, LEN, and Atez/Bev groups. The results were expressed as hazard ratios (HRs) with 95% confidence intervals (CIs), and adjusted survival curves were generated to visualize the findings.

All statistical analyses were performed using the R software (version 4.5.0, R Foundation for Statistical Computing, Vienna, Austria).

## 3 Results

### 3.1 Study Population

A total of 1542 patients were included in the overall cohort, with 823 patients in Era 1, 98 in Era 2, 369 in Era 3, and 252 in Era 4. The median follow‐up period for the overall cohort was 34.9 months (95% CI, 32.3–40.8). Median follow‐up according to treatment era was 68.9 months (95% CI, 52.1–83.3) in Era 1, 61.0 months (95% CI, 32.9–75.4) in Era 2, 42.2 months (95% CI, 40.1–47.1) in Era 3, and 25.7 months (95% CI, 24.6–27.5) in Era 4. The baseline characteristics of the overall cohort are summarized in Table 1. Several temporal trends in the patients' background were observed. The median age increased significantly from 70 years in Era 1 to74 years in Era 4 (*p* < 0.001). The proportion of patients with non‐viral liver disease increased from 32.2% in Era 1% to 8.3% in Era 4 (*p* < 0.001). No marked differences were observed in hepatic reserve parameters, including Child–Pugh class and ALBI grade. The proportion of patients with alpha‐fetoprotein levels exceeding 400 ng/mL decreased from 43.0% in Era 1% to 29.1% in Era 4 (*p* < 0.001).

**TABLE 1:** Demographic and baseline characteristics of 1542 hepatocellular carcinoma patients who received systemic therapy.

Characteristic	Era1 *n* = 823	Era2 *n* = 98	Era3 *n* = 369	Era4 *n* = 252	*p*
Sex, male	664 (80.7%)	73 (74.5%)	297 (80.5%)	210 (83.3%)	0.314
Age, median (IQR)	70 [22, 89]	72 [45, 92]	72 [40, 88]	74 [39, 91]	< 0.001
ECOG PS 0	674 (82.1%)	81 (83.5%)	290 (78.6%)	212 (84.5%)	0.182
Background liver disease					
HBV	132 (16.0%)	16 (16.3%)	45 (12.2%)	35 (13.9%)	< 0.001
HCV	421 (51.2%)	43 (43.9%)	147 (39.8%)	70 (27.8%)	
HBV + HCV	5 (0.6%)	0	7 (1.9%)	0	
Non‐viral	265 (32.2%)	39 (39.8%)	170 (46.1%)	147 (58.3%)	
Child‐Pugh class					
A	718 (87.3%)	85 (86.7%)	311 (84.3%)	212 (84.8%)	0.503
B	102 (12.4%)	12 (12.2%)	56 (15.2%)	38 (15.2%)	
C	2 (0.2%)	1 (1.0%)	1 (0.3%)	0	
ALBI grade					
1	266 (32.4%)	29 (29.6%)	115 (31.2%)	86 (34.3%)	0.805
2	539 (65.6%)	67 (68.4%)	236 (64.0%)	157 (62.5%)	
3	17 (2.1%)	2 (2.0%)	18 (4.9%)	8 (3.2%)	
BCLC stage					
A	9 (1.1)	2 (2.0)	20 (5.4)	7 (2.8)	< 0.001
B	251 (30.5)	26 (26.5)	135 (36.6)	85 (33.7)	
C	561 (68.2)	69 (70.4)	211 (57.2)	160 (63.5)	
D	2 (0.2)	1 (1.0)	3 (0.8)	0	
MVI present	284 (34.6%)	33 (33.7%)	101 (27.4%)	75 (29.9%)	0.078
EHS present	317 (38.7%)	34 (34.7%)	117 (31.8%)	92 (36.5%)	0.151
AFP > 400 ng/mL	354 (43.0%)	34 (34.7%)	117 (31.7%)	73 (29.1%)	< 0.001
First line regimen					
Sorafenib	823 (100.0%)	96 (98.0%)	76 (20.6%)	12 (4.8%)	< 0.001
Lenvatinib	0	2 (2.0%)	293 (79.4%)	30 (11.9%)	
Atezolizumab plus Bevacizumab	0	0	0	210 (83.3%)	

Abbreviations: AFP, alpha‐fetoprotein; ALBI, Albumin‐Bilirubin; BCLC, Barcelona Clinic Liver Cancer; ECOG, Eastern Cooperative Oncology Group; EHS, Extrahepatic spread; HBV, hepatitis B virus; HCV hepatitis C virus; IQR, interquartile range; MVI, Macrovascular invasion; PS, performance status.

The main analysis cohort was defined by excluding patients from Era 1, when no standard post‐treatment options were available, those who received concomitant or inter‐line locoregional therapy, and those enrolled in clinical trials after first‐line treatment. This cohort consisted of 612 patients, including 168 in the SOR group, 264 in the LEN group, and 180 in the Atez/Bev group. The median follow‐up period in the main analysis cohort was 28.3 months (95% CI, 26.7–32.3). Median follow‐up by regimen was 44.2 months (95% CI, 32.8–57.7) in the SOR group, 38.2 months (95% CI, 26.5–42.9) in the LEN group, and 26.7 months (95% CI, 24.6–27.9) in the Atez/Bev group. The baseline characteristics of the study cohort are presented in Table 2. Age and sex distributions were comparable among the three treatment groups. However, patients in the Atez/Bev group more frequently had ECOG PS 0 (86.1%, *p* = 0.011) and Child–Pugh class A (88.3%, *p* = 0.012), suggesting a relatively preserved performance status and liver function. Non‐viral etiology was also most common in the Atez/Bev group (53.9%, *p* = 0.042). Tumor‐related characteristics, including disease stage, macrovascular invasion, extrahepatic spread, and proportion of patients with AFP ≥ 400 ng/mL, were broadly similar across the treatment groups.

**TABLE 2:** Demographic and baseline characteristics of 612 hepatocellular carcinoma patients who received systemic therapy (Main‐analysis Cohort).

Characteristic	Sorafenib *n* = 168	Lenvatinib *n* = 264	Atezolizumab + Bevacizumab *n* = 180	*p*
Sex, male	128 (76.2%)	208 (78.8%)	153 (85.0%)	0.102
Age, median (IQR)	74 [45, 92]	73 [40, 88]	74 [39, 89]	0.169
ECOG PS 0	135 (80.8%)	196 (74.8%)	155 (86.1%)	0.011
Background liver disease				
HBV	20 (11.9%)	34 (12.9%)	24 (13.3%)	0.042
HCV	0	7 (2.7%)	0	
HBV + HCV	70 (41.7%)	103 (39.0%)	59 (32.8%)	
Non‐viral	78 (46.4%)	120 (45.5%)	97 (53.9%)	
Child‐Pugh class				
A	129 (76.8%)	222 (84.7%)	158 (88.3%)	0.013
B	37 (22.0%)	40 (15.3%)	21 (11.7%)	
C	2 (1.2%)	0	0	
ALBI grade				
1	45 (26.8%)	80 (30.4%)	67 (37.2%)	0.074
2	111 (66.1%)	174 (66.2%)	108 (60.0%)	
3	12 (7.1%)	9 (3.4%)	5 (2.8%)	
BCLC stage				
A	10 (6.0%)	11 (4.2%)	3 (1.7%)	0.34
B	55 (32.7%)	87 (33.0%)	57 (31.7%)	
C	101 (60.1%)	164 (62.1%)	120 (66.7%)	
D	2 (1.2%)	2 (0.8%)	0	
MVI present	40 (23.8%)	84 (31.8%)	58 (32.4%)	0.137
EHS present	56 (33.3%)	96 (36.4%)	64 (35.6%)	0.810
AFP > 400 ng/mL	52 (31.0%)	101 (38.4%)	53 (29.4%)	0.099

Abbreviations: AFP, alpha‐fetoprotein; ALBI, Albumin‐Bilirubin; BCLC, Barcelona Clinic Liver Cancer; ECOG, Eastern Cooperative Oncology Group; EHS, Extrahepatic spread; HBV, hepatitis B virus; HCV hepatitis C virus; IQR, interquartile range; MVI, Macrovascular invasion; PS, performance status.

### 3.2 Survival Outcomes and Tumor Response in the Overall and Main Analysis Cohorts

Figure S2 depicts the Kaplan–Meier curves for OS, PFS, and PPS across the four eras in the entire cohort. Median OS improved significantly over time, increasing from 11.5 months (95% CI, 10.2–12.7) in Era 1 to 22.4 months (95% CI, 18.0–26.5) in Era 4 (*p* < 0.001). The median PFS also lengthened from 2.8 months (95% CI, 2.7–3.1) in Era 1 to 6.9 months (95% CI, 5.4–8.3) in Era 4 (*p* < 0.001). In contrast, PPS demonstrated only modest improvement, with medians of 8.2 months (95% CI, 7.3–9.1) in Era 1, 11.3 months (95% CI, 8.1–16.7) in Era 2, 8.0 months (95% CI, 6.7–9.8) in Era 3, and 10.4 months (95% CI, 8.4–12.6) in Era 4 (*p* = 0.038).

**FIGURE 1:**
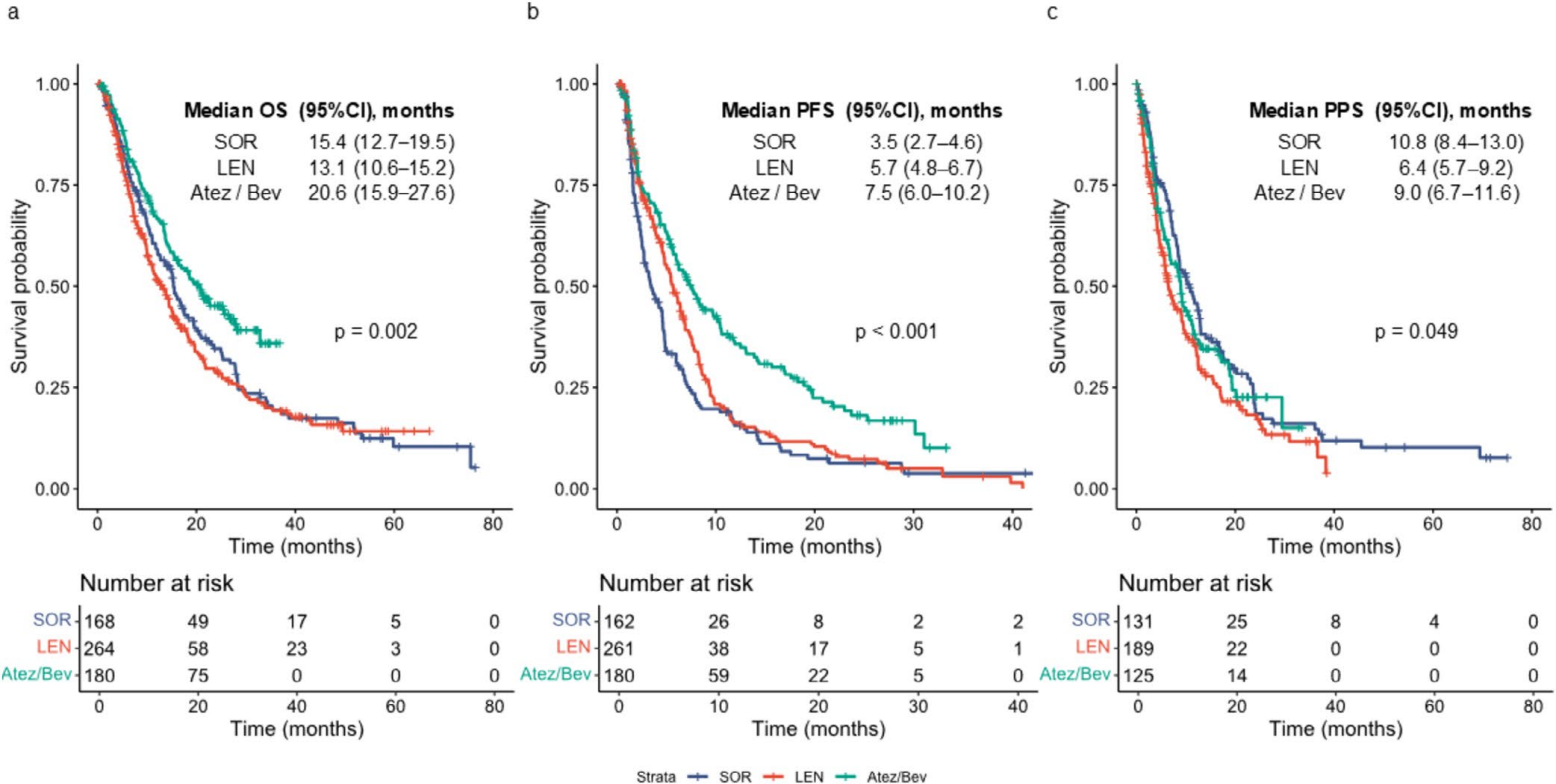
Kaplan–Meier estimates of OS, PFS, and PPS in the main analysis cohort. (a) OS, (b) PFS, and (c) PPS stratified by first‐line treatment regimen (sorafenib, lenvatinib, or atezolizumab plus bevacizumab). OS, overall survival; PFS, progression‐free survival; PPS, post‐progression survival.

Kaplan–Meier curves for OS, PFS, and PPS in the main analysis cohort are shown in Figure 1. Median OS was 15.4 months (95% CI, 12.2–19.4) with SOR, 13.1 months (95% CI, 10.3–15.2) with LEN, and 20.6 months (95% CI, 15.4–26.5) with Atez/Bev (*p* = 0.002). Median PFS was 3.5 months (95% CI, 2.7–4.6), 5.7 months (95% CI, 4.8–6.5), and 7.5 months (95% CI, 6.0–10.1), respectively (*p* < 0.001). Median PPS was 10.8 months (95% CI, 8.4–12.9), 6.4 months (95% CI, 5.3–9.1), and 9.0 months (95% CI, 6.5–11.2), respectively (*p* = 0.049). In the intention‐to‐treat analysis, the overall response rates (ORR) were 7.1% for SOR, 24.2% for LEN, and 24.4% for Atez/Bev (*p* < 0.001). The disease control rates (DCR) were 50.0%, 65.2%, and 74.4%, respectively (*p* < 0.001; Table S1).

### 3.3 Time‐Course of Treatment Status Following First‐Line Therapy

**FIGURE 2:**
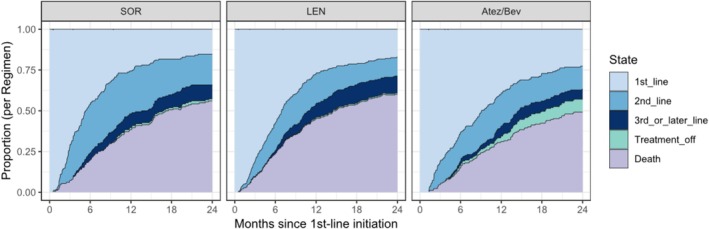
Changes in treatment status following first‐line therapy, illustrated using stacked area plots over a 2‐year period. Patients were categorized into first‐line continuation, second‐line therapy, third‐line or later therapy, treatment off, or death. SOR, sorafenib; LEN, lenvatinib; Atez/Bev, atezolizumab plus bevacizumab.

Beyond conventional survival endpoints, we examined longitudinal treatment dynamics. The time‐course of treatment status was illustrated using stacked area plots for each regimen (Figure 2). Patients were categorized into the following treatment statuses: ongoing first‐line therapy, second‐line therapy, third‐ or later‐line therapy, treatment‐off, and death. Their proportions were tracked over a 2‐year period after treatment initiation.

In the SOR group, an early transition to second‐ or later‐line therapy was frequently observed, with a substantial proportion of patients progressing to death at an early stage. Early transitions were also noted in the LEN group; however, the proportion remaining in second‐line therapy was slightly lower than that in the SOR group, while the proportion progressing to death was similarly high. In contrast, the Atez/Bev group exhibited a more gradual shift, with a notable proportion of patients maintaining first‐line therapy for extended periods or achieving treatment‐off following response. These time‐course patterns clearly differed from those observed in the SOR and LEN groups.

### 3.4 Duration of Disease Control, Duration of Response, and Their Prognostic Impact

To clarify not only the magnitude but also the durability of treatment benefit, we analyzed the duration of disease control (DDC) and duration of response (DOR) in first‐line therapy across regimens and further assessed their prognostic relevance using time‐dependent Cox regression.

**FIGURE 3:**
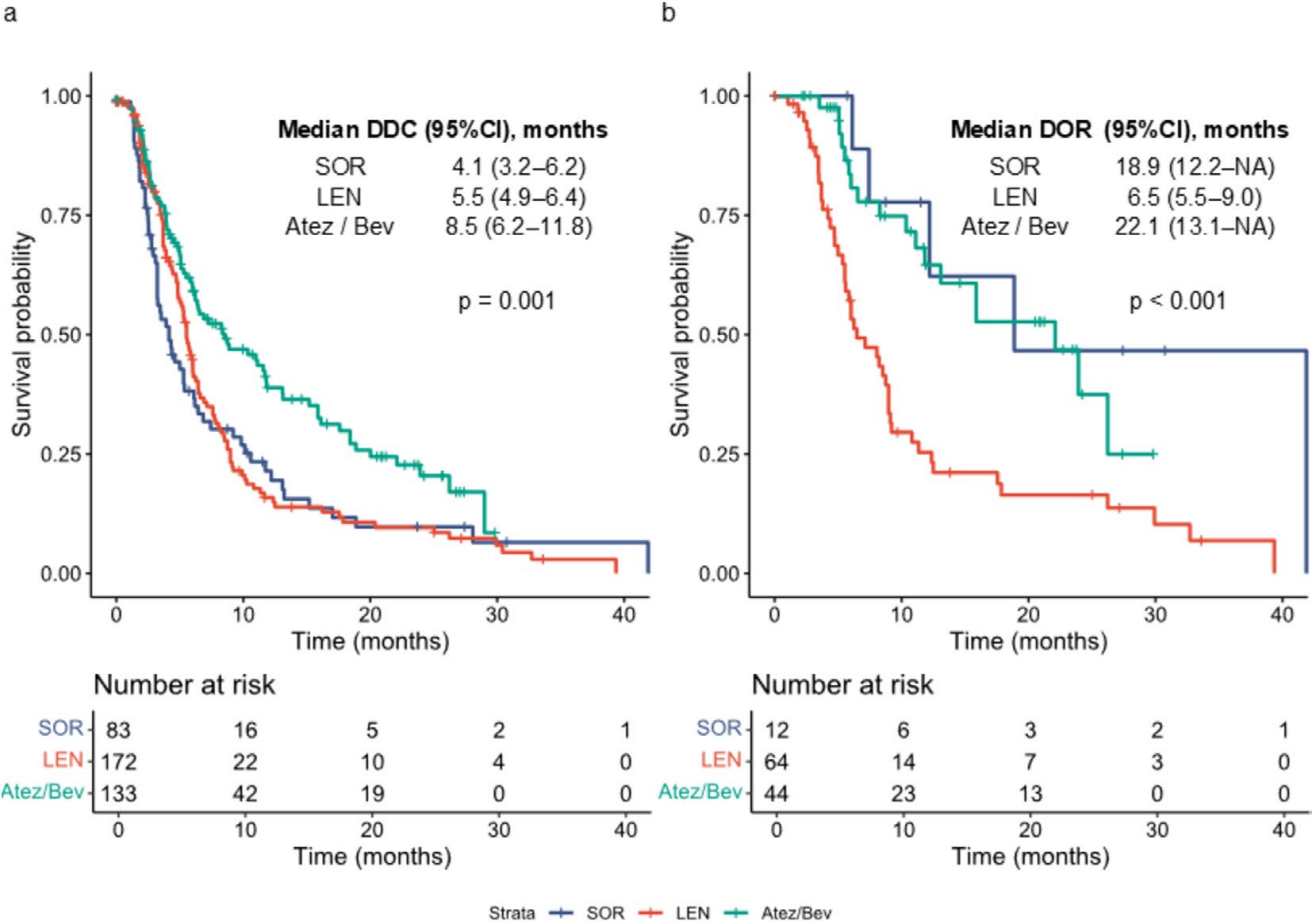
Comparison of DDC and DOR among regimens. Kaplan–Meier estimates are shown for (a) DDC and (b) DOR. DDC, duration of disease control; DOR, duration of response; SOR, sorafenib; LEN, lenvatinib; Atez/Bev, atezolizumab plus bevacizumab.

The median DDC and DOR by regimen are shown in Figure 3. Median DDC was 4.1 months (95% CI, 3.2–5.4) for SOR, 5.5 months (95% CI, 4.8–6.0) for LEN, and 8.5 months (95% CI, 6.0–11.8) for Atez/Bev (*p* = 0.001), with Atez/Bev achieving the longest duration. Median DOR was 18.9 months (95% CI, 6.1–NA) for SOR, 6.5 months (95% CI, 5.5–9.0) for LEN, and 22.1 months (95% CI, 11.1–NA) for Atez/Bev (*p* < 0.001). The notably prolonged DDC and DOR with Atez/Bev highlight its potential for sustained tumor control. Interpretation of the SOR group requires caution because of the small number of responders (*n* = 12) and wide confidence intervals, including NA.

**FIGURE 4:**
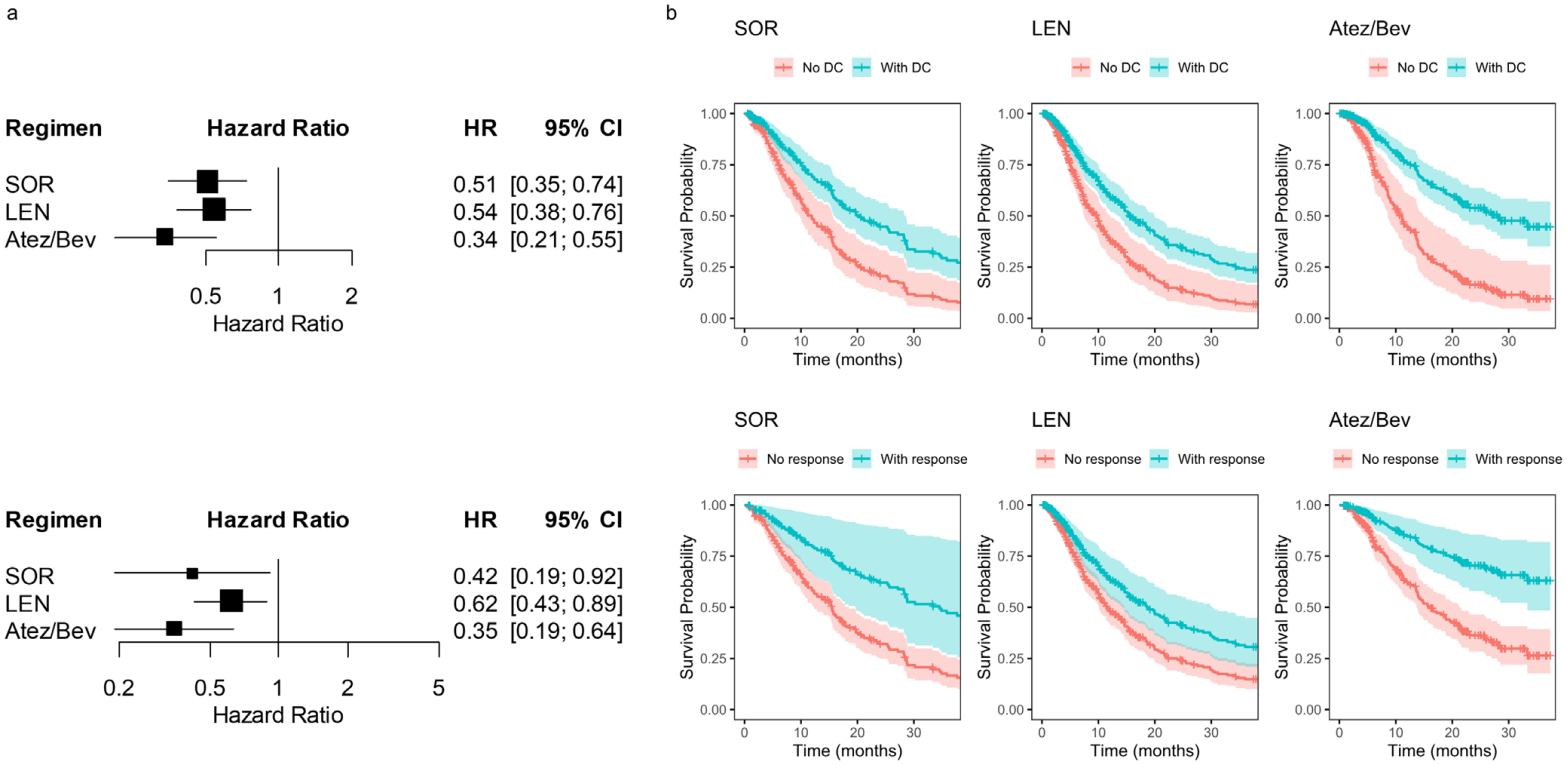
Prognostic impact of achieving disease control or tumor response on overall survival assessed by time‐dependent Cox regression. (a) Adjusted HRs for OS according to achievement of disease control (upper panel) and tumor response (lower panel) in the SOR, LEN, and Atez/Bev groups. (b) Adjusted survival curves for OS stratified by achievement of disease control (upper panel) and tumor response (lower panel). OS, overall survival; HRs, hazard ratios; CI, confidence interval; SOR, sorafenib; LEN, lenvatinib; Atez/Bev, atezolizumab plus bevacizumab.

To determine whether achieving disease control or objective response translated into improved long‐term outcomes, we conducted time‐dependent Cox regression analysis (Figure 4A). Achieving disease control was significantly associated with better OS in all regimens, with HR 0.51 (95% CI, 0.35–0.74; *p* = 4.81 × 10^−4^) for SOR, 0.54 (95% CI, 0.38–0.76; *p* = 4.18 × 10^−4^) for LEN, and 0.34 (95% CI, 0.21–0.55; *p* = 6.77 × 10^−6^) for Atez/Bev. Similarly, achieving an objective response was associated with favorable OS, with HR 0.42 (95% CI, 0.19–0.91; *p* = 0.028) for SOR, 0.62 (95% CI, 0.43–0.89; *p* = 0.010) for LEN, and 0.35 (95% CI, 0.19–0.63; *p* = 0.00048) for Atez/Bev. In both analyses, the strongest prognostic association was observed in the Atez/Bev group. The adjusted survival curves generated from the time‐dependent Cox model (Figure 4B) visually confirmed these associations.

### 3.5 Treatment Sequences and Outcomes Following First‐Line Therapy

**FIGURE 5:**
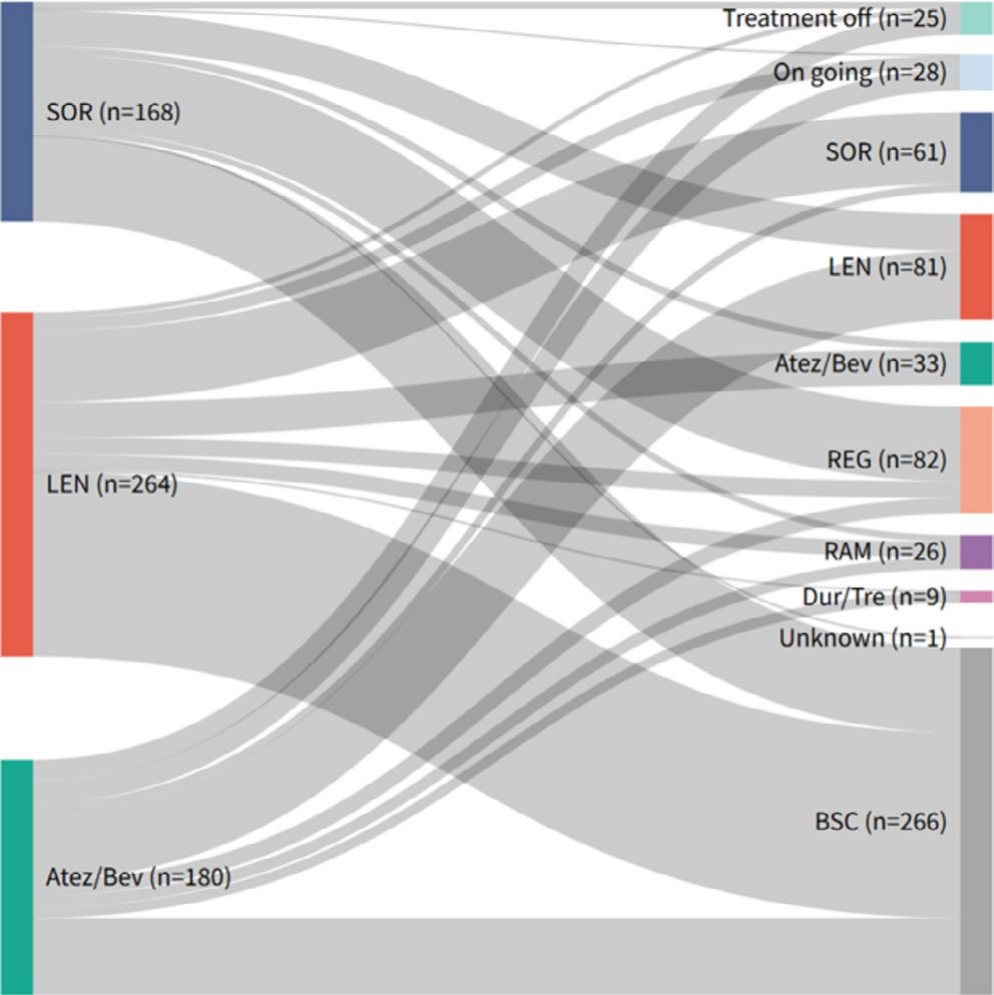
Treatment flow following first‐line therapy, illustrated by a Sankey diagram. The diagram shows patient transitions from first‐line regimens to BSC, second‐line systemic therapy, treatment off, or conversion therapy. BSC, best supportive care; SOR, sorafenib; LEN, lenvatinib; Atez/Bev, atezolizumab plus bevacizumab; REG, regorafenib; RAM, ramucirumab; CAB, cabozantinib; Dur/Tre, durvalumab plus tremelimumab; BSC, best supportive care.

The treatment flow after first‐line therapy initiation is illustrated in the Sankey diagram (Figure 5). First‐line regimens included SOR, LEN, and Atez/Bev, with subsequent pathways involving transition to best supportive care (BSC), second‐line systemic therapy, treatment off, or conversion therapy. The most frequent treatment sequences were SOR–REG (*n* = 57), LEN–SOR (*n* = 55), and Atez/Bev–LEN (*n* = 53). Notably, in the Atez/Bev group, a subset of patients achieved conversion therapy (*n* = 8) or treatment‐off (*n* = 8) as a result of favorable responses, which were rarely observed in the SOR and LEN groups.

**FIGURE 6:**
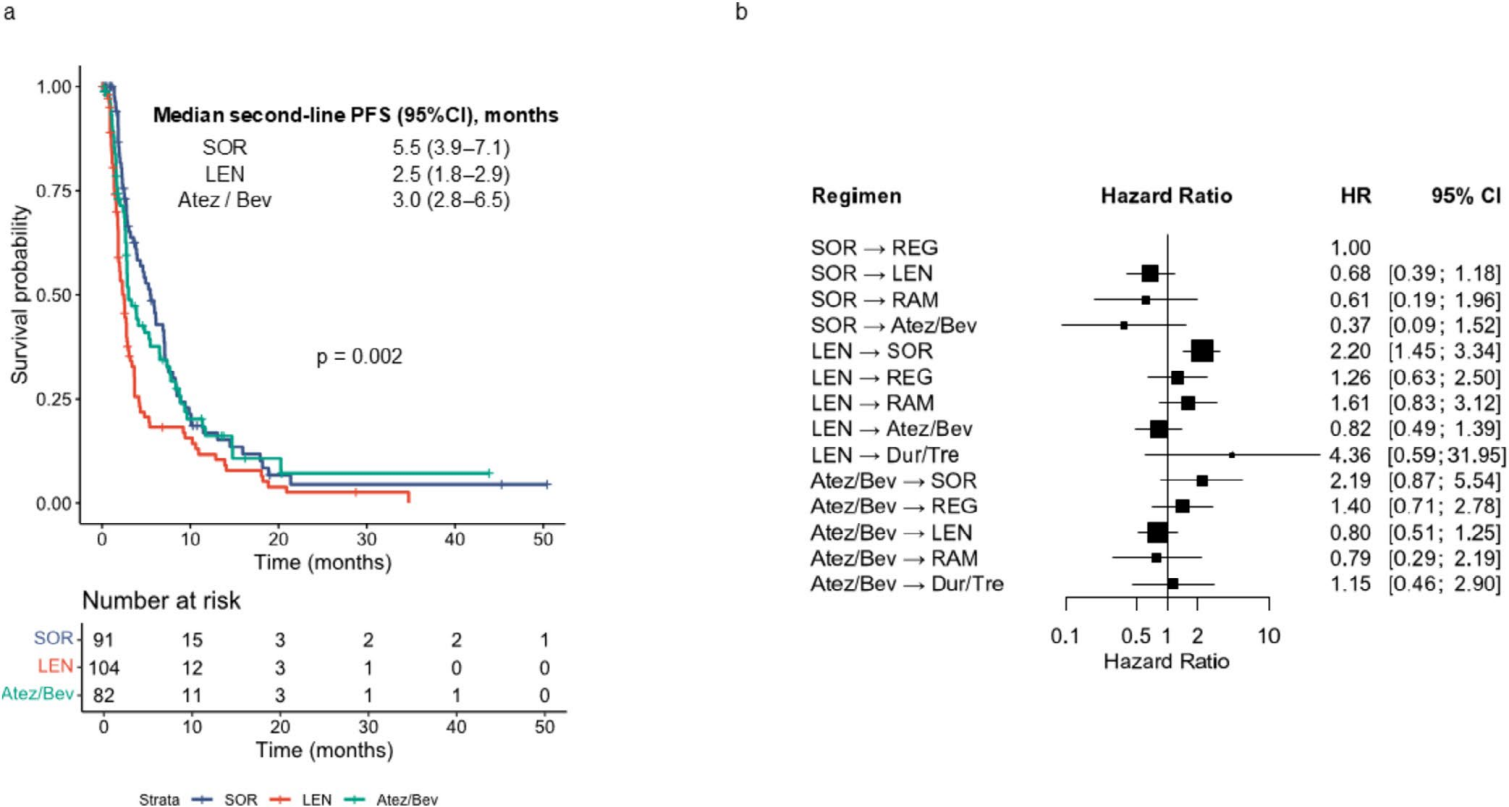
Comparative analysis of PFS after second‐line therapy. (a) Kaplan–Meier estimates of second‐line PFS stratified by first‐line regimen. (b) Hazard ratios for PFS across different treatment sequences, with sorafenib followed by regorafenib as the reference sequence. PFS, progression‐free survival; HR, hazard ratio; CI, confidence interval; SOR, sorafenib; LEN, lenvatinib; Atez/Bev, atezolizumab plus bevacizumab; REG, regorafenib; RAM, ramucirumab; CAB, cabozantinib; Dur/Tre, durvalumab plus tremelimumab.

To examine the outcomes of these treatment flows, we compared progression‐free survival (PFS) after the initiation of second‐line therapy according to the first‐line regimen and treatment sequence (Figure 6). Median second‐line PFS was 5.5 months (95% CI, 3.81–7.04) after SOR, 2.5 months (95% CI, 1.84–2.76) after LEN, and 3.0 months (95% CI, 2.76–5.46) after Atez/Bev (*p* = 0.001) (Figure 6A). In the sequence‐based Cox analysis using SOR–REG as the reference, LEN–SOR showed significantly shorter PFS (HR 2.20, 95% CI, 1.45–3.34). Other sequences did not differ significantly from SOR–REG, although some regimens demonstrated comparable or numerically favorable outcomes, including SOR–LEN (median 7.1 months, 95% CI, 4.90–10.13; HR 0.68, 95% CI, 0.39–1.18), LEN–Atez/Bev (median 3.1 months, 95% CI, 2.56–14.07; HR 0.82, 95% CI, 0.49–1.39), and Atez/Bev–LEN (median 4.1 months, 95% CI, 2.80–9.40; HR 0.80, 95% CI, 0.51–1.25) (Figure 6B; Table S2).

To investigate the factors contributing to limited post‐progression outcomes, we assessed patient status at the time of progressive disease (PD) of first‐line treatment and before the initiation of second‐line treatment. In the analysis of the main analysis cohort at PD (Table S3), liver function deteriorated notably in the Atez/Bev group during first‐line treatment. Specifically, the proportion of Child‐Pugh class A patients decreased from 88.3% at baseline to 64.8% at PD, and the proportion of ALBI grade 1 patients dropped from 37.2% to 19.2%. In contrast, the SOR group showed a relatively modest decline (Child‐Pugh class A: 76.8%–64.1%; ALBI grade 1: 26.8%–26.0%).

Conversely, among patients who successfully transitioned to second‐line therapy (Table 3), there were no significant differences in liver function (Child‐Pugh class, ALBI grade) or tumor burden (BCLC stage, AFP) before the initiation of second‐line treatment. Furthermore, the reasons for discontinuation of second‐line treatment, including radiological progression and adverse events–were comparable across the groups (*p* = 0.304).

**TABLE 3:** Patient status at transition to second‐line therapy and subsequent treatment outcomes (Main‐analysis Cohort).

Characteristic	Sorafenib *n* = 96	Lenvatinib *n* = 108	Atezolizumab + Bevacizumab *n* = 88	*p*
Child‐Pugh class				
A	73 (78.5%)	79 (73.1%)	67 (76.1%)	0.542
B	18 (19.4%)	26 (24.1%)	21 (23.9%)	
C	2 (2.2%)	3 (2.8%)	0	
ALBI grade				
1	31 (32.3%)	34 (31.5%)	22 (25.3%)	0.723
2	58 (60.4%)	65 (60.2%)	60 (69.0%)	
3	7 (7.3%)	9 (8.3%)	5 (5.7%)	
BCLC stage				
B	38 (39.6%)	36 (33.6%)	31 (35.2%)	0.666
C	58 (60.4%)	71 (66.4%)	57 (64.8%)	
AFP > 400 ng/mL	32 (34.0%)	36 (33.6%)	33 (37.9%)	0.798
Reason for discontinuation of second‐line treatment				
PD	61 (63.5%)	83 (76.9%)	57 (64.8%)	0.304
AE	25 (26.0%)	20 (18.5%)	22 (25.0%)	
Other	7 (7.3%)	5 (4.6%)	7 (8.0%)	
Ongoing	3 (3.1%)	0	2 (2.3%)	

Abbreviations: AE, adverse event; AFP, alpha‐fetoprotein; ALBI, Albumin‐Bilirubin; BCLC, Barcelona Clinic Liver Cancer; PD, progressive disease.

## 4 Discussion

This multicenter retrospective cohort study, encompassing 1542 patients with advanced HCC, was distinguished by its direct comparison of SOR, LEN, and Atez/Bev across different treatment eras. Unlike previous retrospective reports, our analysis incorporated era‐based temporal comparisons, a rigorously defined main analysis cohort, and advanced statistical approaches, including time‐dependent Cox regression and longitudinal treatment course evaluations. These methodological strengths enabled a multidimensional characterization of how the introduction of immunotherapy qualitatively transformed treatment dynamics in advanced HCC. By applying this comprehensive framework, we were able to clarify two principal issues that have not been fully addressed in prior studies.

First, Atez/Bev treatment significantly prolonged OS, PFS, and DCR compared to VEGF‐TKIs. In the era‐based analysis, median OS improved from 11.5 months in Era 1 (sorafenib monotherapy era) to 22.4 months in Era 4 (Atez/Bev era), while median PFS increased from 2.8 to 6.9 months. In the regimen‐based analysis, the Atez/Bev group achieved a median OS of 20.6 months and a median PFS of 7.5 months, both exceeding those of SOR and LEN. Moreover, Atez/Bev yielded the longest median DDC (8.5 months) and DOR (22.1 months), underscoring its ability to provide sustained tumor control. These findings corroborate post hoc analyses of IMbrave150, showing that the depth and durability of tumor control are strong predictors of OS [11, 26]. Our time‐dependent Cox regression further confirmed that achieving disease control or response was significantly associated with improved OS across all regimens, with the strongest effect observed in the Atez/Bev group. In addition, longitudinal treatment‐course analysis revealed that a subset of patients in the Atez/Bev group achieved treatment‐off or curative conversion, outcomes rarely observed in the TKI era, illustrating how Atez/Bev has introduced a qualitatively new therapeutic paradigm. Our data therefore extends the post hoc findings of IMbrave150 by validating the prognostic importance of treatment durability in a large multicenter real‐world cohort, while also highlighting distinct treatment trajectories such as treatment‐off and conversion. In contrast to registry studies, including the Asia–Pacific cohort [21], the Japanese HERITAGE study [27], and the European AB‐Real study [28], which have confirmed the reproducibility of Atez/Bev efficacy but provided limited insight into underlying dynamics, our analysis uniquely integrates era‐based comparisons, regimen‐specific outcomes, and time‐dependent Cox modeling. This comprehensive approach allowed us to not only confirm existing observations but also expand them, yielding new insights into the durability and prognostic impact of disease control.

Beyond conventional survival outcomes, our study underscores the clinical significance of treatment durability as a defining feature of Atez/Bev. While post hoc analyses of IMbrave150 first suggested that the depth and duration of response strongly predict OS [11, 26], our data extend this concept by demonstrating, in a large multicenter real‐world cohort, that prolonged DDC and DOR translate into long‐term survival and distinct treatment trajectories, including treatment‐off and curative conversion. Importantly, our findings highlight the heterogeneous nature of the benefits of Atez/Bev; although median OS and PFS improved substantially at the population level, the most remarkable long‐term outcomes were essentially confined to patients achieving deep or durable responses. This two‐phase pattern of benefit, often obscured by median values, emphasizes the need for novel endpoints, such as DOR, DDC, or shrinkage‐stable disease, which may more accurately capture the therapeutic potential of immunotherapy in HCC.

Our results are broadly consistent with those reported in international registries [4, 21, 27], which collectively confirmed the reproducible efficacy of Atez/Bev in routine practice. However, our analysis was unique in several respects. By integrating era‐based comparisons, regimen‐specific outcomes, and time‐dependent Cox models, we were able to clarify not only the magnitude but also the durability and prognostic impact of disease control, thereby complementing the findings of clinical trials. Furthermore, by evaluating treatment sequences, we provided real‐world evidence that extends beyond existing registry data, which have so far offered limited insight into post‐Atez/Bev outcomes. Taken together, these features distinguish our study as an important complement to clinical trials and international registries, bridging pivotal trial findings with everyday practice and highlighting the evolving therapeutic paradigm introduced by Atez/Bev.

Second, the post‐progression outcomes after Atez/Bev remained suboptimal. In our cohort, the median PPS in the Atez/Bev group was 9.0 months, essentially unchanged from the 10.8 months observed with SOR. Among patients receiving second‐line therapy, median PFS was only 3.0 months after Atez/Bev, compared with 5.5 months after SOR and 2.5 months after LEN. Notably, the Atez/Bev–LEN sequence yielded relatively favorable PFS (4.1 months), whereas sequences such as Atez/Bev–SOR or LEN–SOR resulted in extremely short PFS (< 2 months). These findings are broadly consistent with prior international series and meta‐analyses reporting PFS of 3–5 months for LEN after Atez/Bev but only 2–3 months for other TKIs such as SOR, LEN, REG, or CAB [19, 20, 21, 22, 23, 24, 28, 29].

The limited efficacy of second‐line therapy is likely multifactorial. To investigate the contributing factors, we assessed liver function at the time of PD and patient status before the initiation of second‐line treatment. First, deterioration of liver function during prolonged first‐line therapy may pose a barrier to subsequent treatment. In our analysis of the entire cohort at PD (Table S3), liver function deteriorated notably in the Atez/Bev group during first‐line treatment. Specifically, the proportion of Child‐Pugh class A patients decreased from 88.3% at baseline to 64.8% at PD, and the proportion of ALBI grade 1 patients dropped from 37.2% to 19.2%. In contrast, the SOR group showed a relatively modest decline (Child‐Pugh class A: 76.8%–64.1%; ALBI grade 1: 26.8%–26.0%). This aligns with accumulating evidence; Kim et al. reported distinct liver function decline in patients treated with Atez/Bev for over a year [30], Celsa et al. highlighted hepatic decompensation as a major driver of mortality [31], and Shimose et al. demonstrated that deterioration of liver function disturbs sequential therapy [32]. These data suggest that liver function deterioration during Atez/Bev treatment filters out candidates for subsequent therapy.

Furthermore, even among patients who maintained eligibility for second‐line therapy, survival outcomes remained poor. Our analysis of the second‐line cohort (Table 3) revealed no significant differences in liver function (Child‐Pugh class, ALBI grade) or tumor burden (BCLC stage, AFP) before the initiation of second‐line treatment among the groups. Furthermore, the reasons for discontinuation of second‐line treatment, including radiological progression and adverse events—were comparable (*p* = 0.304). This suggests that the limited PPS is not solely due to patient frailty but may also reflect reduced efficacy of current second‐line agents following immunotherapy. Mechanistically, adaptive resistance pathways beyond VEGF signaling (such as fibroblast growth factor or mesenchymal–epithelial transition factor activation) and immunosuppressive remodeling of the tumor microenvironment after VEGF blockade may attenuate the activity of subsequent TKIs [33, 34, 35].

Taken together, our results indicate that although LEN appears relatively active in the post‐Atez/Bev setting, overall second‐line efficacy does not clearly exceed that of the SOR–REG era, where a well‐validated sequence had once improved survival. The modest PPS benefit observed with Atez/Bev reflects not only impaired liver function at progression but also the absence of effective therapeutic options capable of overcoming resistance mechanisms. Consequently, future work is required to elucidate these resistance pathways and to develop rational therapeutic strategies, such as upfront combination strategies or novel agents. These limitations highlight the urgent need to establish evidence‐based sequencing frameworks and to identify biomarkers that can guide early switching, particularly in patients with primary resistance. Given that current second‐line choices after Atez/Bev are based on retrospective or small‐scale studies, prospective trials and international registries will be essential to define optimal strategies in this increasingly common clinical scenario.

In conclusion, this large multicenter real‐world study demonstrates that Atez/Bev has fundamentally reshaped the therapeutic landscape of advanced HCC by improving survival through durable tumor control and introducing new treatment trajectories such as treatment‐off and conversion therapy. However, prognosis after Atez/Bev failure remains suboptimal, with PPS outcomes comparable to the TKI era and no clearly established second‐line standard. These findings highlight both the transformative potential and the limitations of Atez/Bev, underscoring the urgent need for optimized sequencing strategies, predictive biomarkers, and prospective trials to guide therapy beyond the first line.

## Author Contributions

**Rui Sato:** conceptualization, methodology, data curation, writing – review and editing. **Masanori Inoue:** conceptualization, methodology, data curation, writing – original draft, writing – review and editing, project administration, visualization, investigation, software, formal analysis, validation. **Sadahisa Ogasawara:** supervision, conceptualization, methodology, writing – original draft, writing – review and editing, project administration, investigation, resources. **Kazuhisa Asahara:** conceptualization, methodology, data curation, writing – review and editing. **Masato Nakamura:** writing – review and editing. **Keisuke Koroki:** writing – review and editing. **Takeshi Aramaki:** writing – review and editing. **Naoya Kanogawa:** writing – review and editing. **Michihisa Moriguchi:** conceptualization, data curation, supervision, writing – review and editing, methodology.

## Funding

The authors have nothing to report.

## Ethics Statement

The study protocol was approved by the ethics committees of all the participating institutions (Chiba University approval number: M10292). The study protocol conformed to the ethical guidelines of the 2013 Declaration of Helsinki, 2018 edition of the Declaration of Istanbul, and was approved by the Research Ethics Committee of the Graduate School of Medicine, Chiba University (No. M10292), and three other institutions (Kyoto Prefectural University of Medicine, Shizuoka Cancer Center, Jichi Medical University). Formal consent by written signatures was not required for this type of study, based on the Ethical Guidelines for Medical and Biological Research Involving Human Subjects in Japan.

## Consent

In accordance with the Japanese Ethical Guidelines for Medical and Health Research Involving Human Subjects, the requirement for written informed consent was waived, and an opt‐out approach was adopted.

## Conflicts of Interest

Sadahisa Ogasawara received honoraria from Eisai, Chugai Pharma, AstraZeneca, and Merck & Co. Inc.; consulting or advisory fees from Eisai, Merck & Co. Inc., Chugai Pharma, and AstraZeneca; and research grants from Bayer, AstraZeneca, Chugai Pharma, and Eisai. Michihisa Moriguchi received honoraria from Eisai, Chugai Pharma, AstraZeneca. Naoki Morimoto received honoraria from AbbVie GK, Eisai, Chugai Pharma; and research grants from AbbVie GK, Eisai. Naoki Morimoto is an editorial board member of Hepatology Research. The other authors have no conflicts of interest to declare. Signed COI disclosure forms for all authors have been submitted by the corresponding author.

## Supporting information


**Data S1:** Supporting Information.

## Data Availability

The data that support the findings of this study are available from the corresponding author upon reasonable request.
