# Supplementary material for: Durable Disease Control With Atezolizumab Plus Bevacizumab but Limited Post‐Progression Outcomes: A Multicenter Real‐World Study in Advanced Hepatocellular Carcinoma
Source: Cancer Med. 2026 Feb 18;15(2):e71655. doi: 10.1002/cam4.71655 (PMC12914342; doi:10.1002/cam4.71655)

***Supplementary Appendix***

This appendix has been provided by the authors to give the reader additional information about their work.

**Supplementary Tables**

**Table S1.** Best overall response according to RECIST criteria in the main analysis cohort by first-line regimen.

| **Response** | **Sorafenib N = 168** | **Lenvatinib N = 264** | **Atezolizumab+ Bevacizumab N = 180** | ***P*-value** |
| --- | --- | --- | --- | --- |
| CR | 1 (0.6%) | 6 (2.3%) | 4 (2.2%) | <0.001 |
| PR | 11 (6.5%) | 58 (22.0%) | 40 (22.2%) |  |
| SD | 72 (42.9%) | 108 (40.9%) | 89 (49.4%) |  |
| PD | 70 (41.7%) | 76 (28.8%) | 42 (23.3%) |  |
| NE | 14 (8.3%) | 16 (6.1%) | 5 (2.8%) |  |
| ORR | 12 (7.1%) | 64 (24.2%) | 44 (24.4%) | <0.001 |
| DCR | 84 (50.0%) | 172 (65.2%) | 133 (73.9%) | <0.001 |

CR, Complete Response; PR, Partial Response; SD, Stable Disease; PD, Progressive Disease; NE, Not Evaluated; ORR, Objective Response Rate; DCR, Disease Control Rate

**Table S2.** Median Progression-Free Survival According to First- and Second-line Regimen Combinations in the Main Analysis Cohort

| **First-line, Second-line** | **n** | **Median PFS (95% CI), months** | **HR (95% CI)** | **p-value** |
| --- | --- | --- | --- | --- |
| 1st: SOR, 2nd: REG | 56 | 4.34 (2.73–7.00) | NA | NA |
| 1st: SOR, 2nd: LEN | 27 | 7.10 (4.93–10.09) | 0.68 (0.39–1.17) | 0.165 |
| 1st: SOR, 2nd: RAM | 3 | 8.38 (3.91–NA) | 0.61 (0.19–1.97) | 0.411 |
| 1st: SOR, 2nd: Atez/Bev | 5 | NA | 0.37 (0.09–1.51) | 0.164 |
| 1st: LEN, 2nd: SOR | 53 | 1.84 (1.74–2.60) | 2.21 (1.46–3.36) | 0.000 |
| 1st: LEN, 2nd: REG | 13 | 2.76 (0.92–NA) | 1.27 (0.64–2.52) | 0.496 |
| 1st: LEN, 2nd: RAM | 12 | 2.65 (2.05–NA) | 1.63 (0.84–3.16) | 0.148 |
| 1st: LEN, 2nd: Atez/Bev | 25 | 3.06 (2.56–14.07) | 0.82 (0.49–1.40) | 0.476 |
| 1st: LEN, 2nd: Dur/Tre | 1 | 1.81 (NA–NA) | 4.53 (0.62–33.30) | 0.138 |
| 1st: Atez/Bev, 2nd: SOR | 5 | 1.45 (1.38–NA) | 2.20 (0.87–5.56) | 0.095 |
| 1st: Atez/Bev, 2nd: REG | 11 | 2.71 (NA–NA) | 1.40 (0.71–2.79) | 0.335 |
| 1st: Atez/Bev, 2nd: LEN | 48 | 4.11 (2.83–9.37) | 0.80 (0.51–1.25) | 0.329 |
| 1st: Atez/Bev, 2nd: RAM | 9 | 2.79 (2.76–NA) | 0.80 (0.29–2.22) | 0.662 |
| 1st: Atez/Bev, 2nd: Dur/Tre | 8 | 2.99 (1.68–NA) | 1.16 (0.46–2.92) | 0.758 |

PFS, progression-free survival; CI, confidence interval; HR, hazard ratio; SOR, Sorafenib; REG, Regorafenib; LEN, Lenvatinib; RAM, Ramucirumab; Atez/Bev, Atezolizumab plus Bevacizumab; Dur/Tre, Durvalumab plus Tremelimumab; NA, not available.

**Table S3.** Liver function at the time of disease progression of first-line treatment (Main-analysis Cohort).

| **Characteristic** | **Sorafenib  n=131** | **Lenvatinib  n=189** | **Atezolizumab + Bevacizumab n=125** | **p.value** |
| --- | --- | --- | --- | --- |
| Child-Pugh class |  |  |  |  |
| A | 84 (64.1) | 111 (58.7) | 81 (64.8) | 0.364 |
| B | 37 (28.2) | 65 (34.4) | 35 (28.0) |  |
| C | 7 ( 5.3) | 12 ( 6.3) | 9 ( 7.2) |  |
| Unknown | 3 ( 2.3) | 1 ( 0.5) | 0 ( 0.0) |  |
| ALBI grade |  |  |  |  |
| 1 | 34 (26.0) | 39 (20.7) | 24 (19.2) | 0.527 |
| 2 | 77 (58.8) | 109 (58.0) | 81 (64.8) |  |
| 3 | 20 (15.3) | 39 (20.7) | 20 (16.0) |  |
| Unknown | 0 ( 0.0) | 1 ( 0.5) | 0 ( 0.0) |  |

ALBI, Albumin-Bilirubin.

**Supplementary Figure Legends**

**Figure S1** Patient selection for the main analysis cohort. SOR, sorafenib; LEN, lenvatinib; Atez/Bev, atezolizumab plus bevacizumab; TACE, transarterial chemoembolization; HAIC, hepatic arterial infusion chemotherapy.

**Figure S2** Kaplan–Meier curves for survival outcomes in the overall cohort. Kaplan–Meier estimates of (a) OS, (b) PFS, and (c) PPS in the entire patient cohort (n=1,542), stratified by treatment era. OS, overall survival; PFS, progression-free survival; PPS, post-progression survival.

**Figure S1**


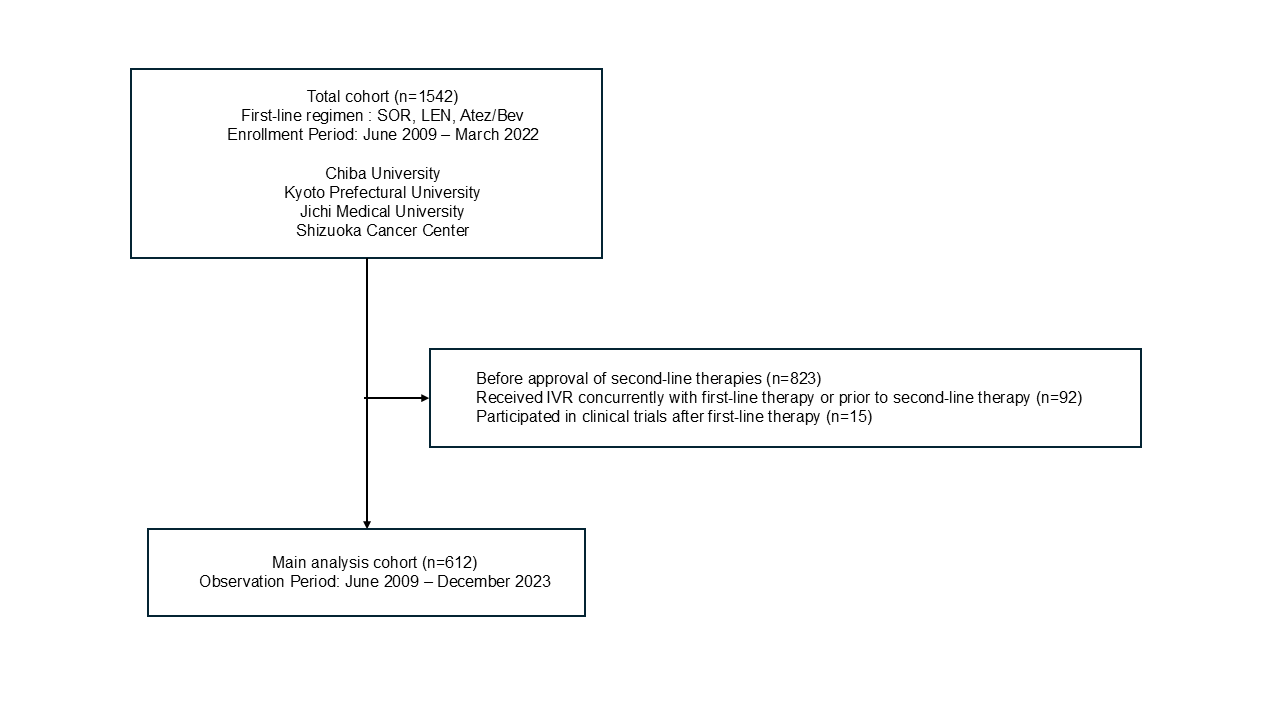
 **Figure S2**
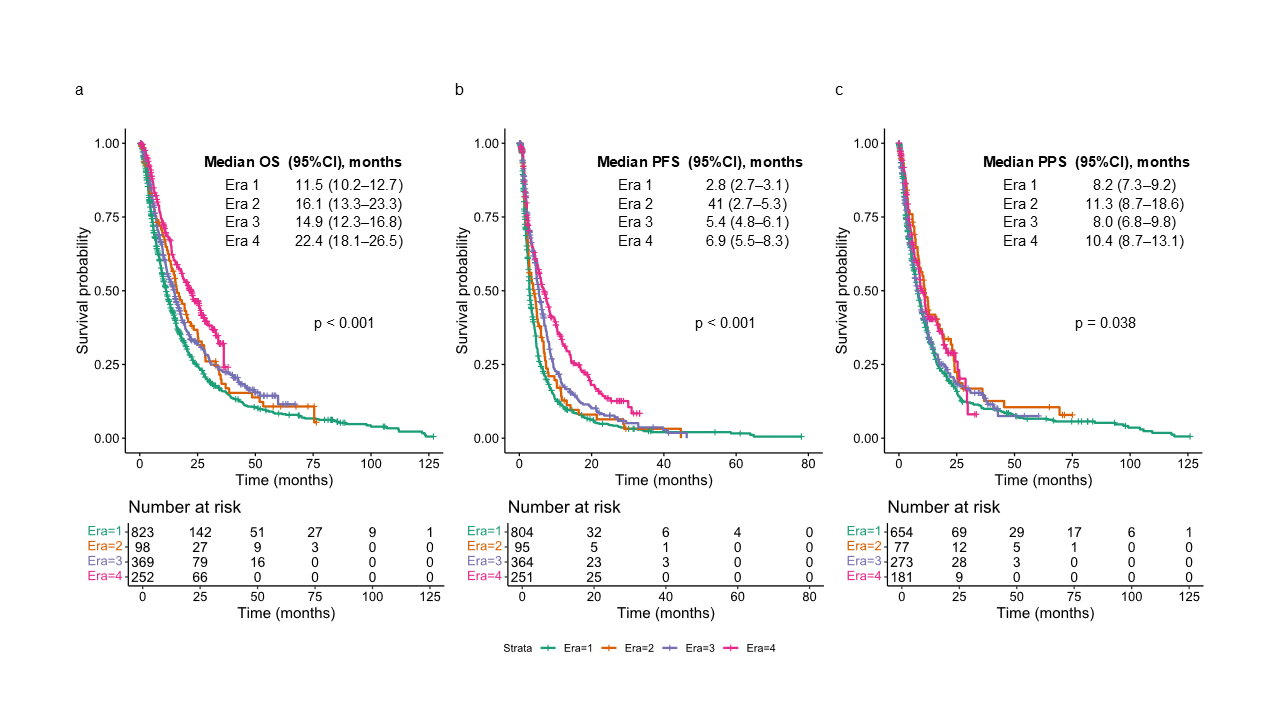

Supplement: Supplementary file 1 — Data S1: Supporting Information. [file CAM4-15-e71655-s001.docx]
